# Proposed Nodal Cancer Index (NCI) in ovarian carcinomatosis

**DOI:** 10.1186/s43046-025-00256-4

**Published:** 2025-04-11

**Authors:** M D Ray, Manish Kumar Gaur

**Affiliations:** Department of Surgical Oncology, DR BRA-IRCH, All India Institute of Medical Sciences, New Delhi, India

**Keywords:** Advanced ovarian cancer, Nodal Cancer Index, Cytoreductive surgery

## Abstract

**Introduction:**

The nodal positivity in advanced ovarian cancers is approximately 68–70% histopathologically. Even after neoadjuvant chemotherapy (NACT) chance of nodal positivity is around 50–80%. In the prevailing literature, the nodal burden is a neglected entity in both assessment and documentation and complete clearance during the CRS. We aim to highlight the importance of nodal dissection and propose a Nodal Cancer Index (NCI) like PCI for ovarian cancers based on our experience of 105 cases.

**Materials and methods:**

We included 105 patients with advanced ovarian cancers who underwent CRS. Retroperitoneal lymph nodes and bilateral pelvic lymph node dissection were routinely done in all the cases. For Nodal Cancer Index calculation, the abdomen is divided into 13 zones, zones 1–6 for retroperitoneum, zones 1–6 for Pelvic nodes, and zone 0 for extra-abdominal nodes. Furthermore, a Nodal size score ranging from 1 to 3 has been proposed so that the Nodal Cancer Index ranges from 13 to 39.

**Results:**

The median age of the patients was 51 years (range 19–71) and the most significant patients were in stage III (65.7%), and 34.3% had stage IV disease at presentation. The lymph nodes were found to be positive in 62 patients (59%), and the positivity rate was higher in patients who underwent upfront surgery 36 (58.1%) as compared to 26 (41.9%) in those who received NACT. The majority of the patients (56.6%) had positive lymph nodes in both the pelvic and retroperitoneal groups, whereas 19.3% had only pelvic nodes positive, and 24.2% had only retroperitoneal nodes positive. The probability of overall survival at 5 years in our patients was 48.9% (95% CI = 35.5–61).

**Conclusion:**

The results of our analytic observation confirm that systemic lymphadenectomy of all 13 zones proposed by our study should be an integral part of optimal CRS in the advanced carcinoma ovary and this will help us manage these advanced cases in a better objective manner.

## Introduction

The incidence of ovarian cancer is rapidly increasing along with breast cancer, and it is considered the most lethal gynecological cancer worldwide [1]. More than two-thirds of the cases are present in advanced stages, i.e., FIGO IIIC onwards. Around the globe, optimal cytoreductive surgery (CRS) followed by systemic chemotherapy is the standard of care for advanced ovarian cancers [2]. In recent times, intraperitoneal chemotherapy like HIPEC has added an essential armamentarium in managing such advanced cases, although optimal cytoreduction is the most prognostic factor.

As per the present concept, CRS includes removing all macroscopic residual disease, but the routine removal of pelvic and retroperitoneal lymph nodes is not an integral part of cytoreduction in the standard of care. The burden of the peritoneal disease is assessed by the Peritoneal Cancer Index (PCI), which also does not include the nodal status. However, in advanced ovarian cancer, nodal involvement is found in 70–75% of cases in pelvic or retroperitoneal nodes or both especially para-aortic, and inter-aortocaval nodes, and as the literature suggests, approximately 68–70% of nodes are positive in advanced ovarian cancer histopathologically. Even after neoadjuvant chemotherapy (NACT) chance of nodal positivity is around 50–80% [3]. In the prevailing literature, the nodal burden is a neglected entity in both assessment and documentation of it and the complete clearance during the CRS. There is ample literature that suggests overall survival is significantly affected by serous histology with positive nodes [4].

This article aims to highlight the importance of nodal dissection and develop a Nodal Cancer Index (NCI) like PCI, based on our experience of 105 cases of systemic nodal dissection in advanced ovarian cancer.

## Materials and methods

The majority of our ovarian cancer cases present in the advanced stage, FIGO stage III and IV, and being a tertiary level referral cancer center, this share reaches more than 90%. A total of 255 ovarian cases were operated at our center between 2012 and 2018, out of which we included 105 advanced ovarian cancers that underwent systemic lymphadenectomy (*n* = 105), and a retrospective analysis was performed from a prospectively maintained computerized database of peritoneal surface malignancies in our center in April 2021.

Inclusion criteria included FIGO stage III and IV, age between 18 and 75 years, ECOG performance status 1 or optimized 2, and patients who underwent complete systemic lymphadenectomy.

Patients in whom significant primary CRS was performed elsewhere, any past pelvic, retroperitoneal surgeries, uncertain and irregular treatment patterns, and history of any past pelvic irradiation were excluded from the study.

Contrast-enhanced computed tomography scans (CECT) of the abdomen and pelvis were performed for preoperative disease burden assessment along with tumor markers CA-125, CEA, and CA 19.9 in all the cases. Based on multidisciplinary tumor board discussion, the patients with poor performance status, multiple comorbidities, CA-125 > 2000, very high radiologic PCI and liver, and spleen parenchymal involvement were given neoadjuvant chemotherapy and reassessed for interval CRS. All other patients underwent 3–4 weeks of pre-habilitation before being taken up for primary CRS.

The surgical procedure included the standard cytoreduction steps, including peritoneal washings in cases of primary CRS, total hysterectomy with bilateral salpingo-oopherectomy, total omentectomy, and multiple quadrant peritoneal biopsies. Bowel resections, disease-specific peritonectomy, and total peritonectomy were performed in selected cases to achieve R-0 resection.

Lymph nodal involvement is part and parcel of advanced ovarian cancer. Without the removal of all nodal burdens in the pelvis as well as the retroperitoneum, we cannot achieve optimal cytoreduction. It also violates the very principles of HIPEC. Without achieving complete cytoreduction, we do not apply HIPEC which is the most evidenced base therapy in ovarian cancer. Therefore, retroperitoneal lymph nodes and bilateral pelvic lymph node dissection were routinely done in all the cases. Optimal cytoreduction was defined as no residual tumor or residual size less than 2.5 mm, as we do HIPEC in selected cases. After the cytoreduction, all patients received adjuvant chemotherapy and were kept on a regular 3-monthly follow-up with history and clinical examination and CA-125 and imaging every 6 months.

The analysis was performed for patients’ and disease characteristics. Optimal cytoreduction rates and overall survival rates were analyzed in both lymph node-positive and negative cohorts. Kaplan Meier was undertaken for survival analysis using STATA version 14.0 (STATA Corp., TX, USA). Overall survival (OS) was calculated from the date of diagnosis until death from any cause.

### Proposed nodal cancer index

The aim of the study is to propose a Nodal scoring system, i.e., the Nodal Cancer Index (NCI) like the Peritoneal Cancer Index (PCI), which is required to stratify the nodal burden and its importance in ovarian cancer management and improving outcomes. We proposed the Nodal Cancer Index in ovarian cancer to better assess and document the nodal burden and improve the management in an objective manner.

**Fig. 1:**
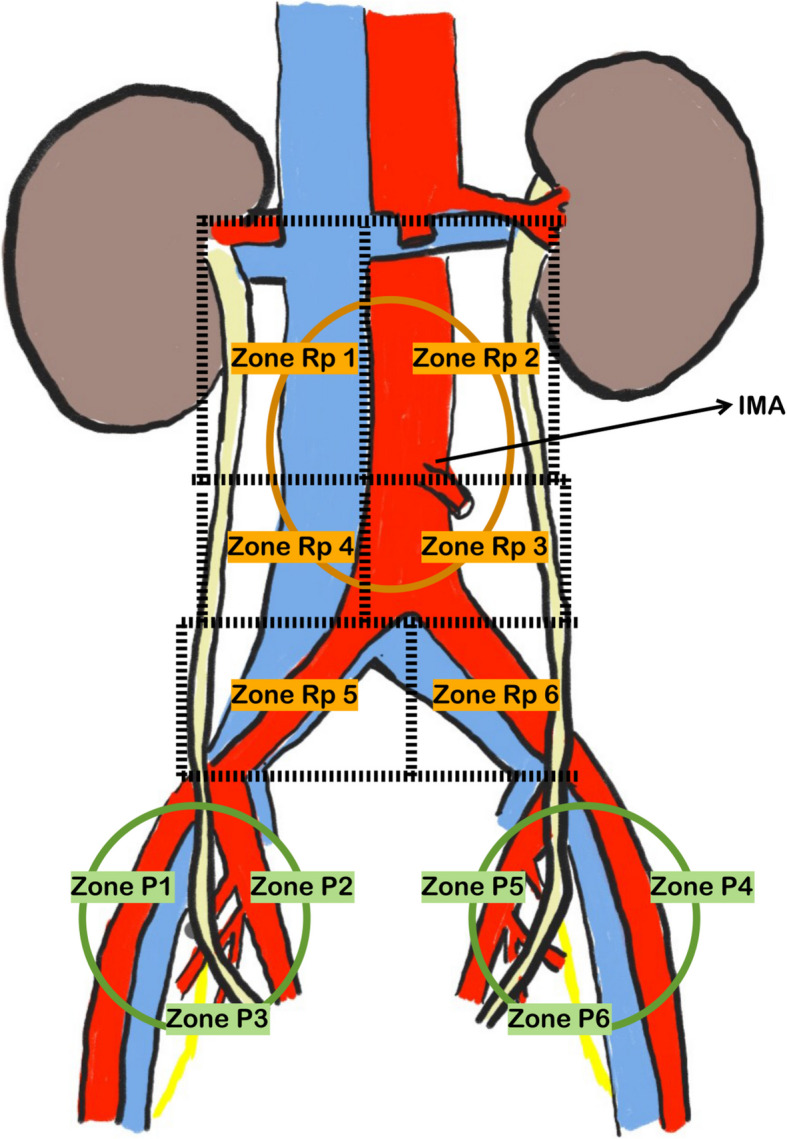
Schematic diagram of the division of retroperitoneal (Rp) and pelvic (P) zones

In the abdomen, we divide the retroperitoneum into six Nodal involvement zones in a clockwise manner [5] (Fig. 1).

#### Zone Rp1

It is bounded superiorly by the right renal vein, an imaginary horizontal line at the level of the inferior mesenteric artery (IMA), the right ureter laterally and medially, and the right border of the aorta.

#### Zone Rp2

It is bounded superiorly by the left renal vein, inferiorly by an imaginary horizontal line at the level of IMA, laterally by the left ureter, and medially by the right border of the aorta.

#### Zone Rp3

This is the space between two imaginary lines. The first one is an imaginary horizontal line at the level of IMA and below the horizontal line at the level of aortic bifurcation, the medially right border of the aorta, and laterally left ureter.

#### Zone Rp4

Like zone 3, it lies between two imaginary lines as in zone Rp3 but the medially right border of the aorta and laterally right ureter.

#### Zone Rp5 and Rp6

This zone is bounded by the horizontal line at the level of aortic bifurcation superiorly, common iliac arteries bifurcations inferiorly on both sides, and laterally on both sides by the ureters. This zone is divided by an imaginary vertical line drawn from the bifurcation of the aorta into right zone 5 and left zone 6.

The pelvis is divided into 6 nodal involvement zones in a clockwise manner.

#### Zone P1

Right external iliac nodes.

#### Zone P2

Right internal iliac nodes.

#### Zone P3

Right obturator nodes.

#### Zone P4

Left external iliac nodes.

#### Zone P5

Left internal iliac nodes.

#### Zone P6

Left obturator nodes.

#### Zone 0

This includes lymph nodes outside the abdominal cavity like supraclavicular nodes, inguinal nodes, axillary nodes, etc.

Total: 13 zones [6 (Rp) + 6 (P) + 1 (zone 0)].

Each region is scored using Nodal Size Score (NSS) as described below:

-  < 1 cm = 1.
- 1–2 cm = 2.
- > 2 cm or conglomerated nodes = 3.
- Maximum score would be 39 [13 Zones × 3 (maximum NSS)], like PCI.

## Results

We *retrospectively* analyzed 105 patients with advanced ovarian cancer who underwent pelvic and retroperitoneal lymph node dissection in this study. The clinicopathological characteristics of the cohort have been depicted in Table 1. The most significant patients were in stage III (65.7%), and 34.3% had stage IV disease at presentation. A total of 60 patients (57.1%) received NACT first, followed by interval CRS and 45 patients (42.9%) underwent primary CRS. The surgical PCI was less than 20 in 88 patients (83.8%) and was more than 20 in 17 patients (16.2%). A CC-0 score could be achieved with our maximal surgical efforts in 83 patients (79%), and a CC-1 score was achieved in 22 patients (21%). The characteristics of lymph node-positive cases have been depicted in Table 2. The lymph nodes were found to be positive in 62 patients (59%), and the positivity rate was higher in patients who underwent upfront surgery 36 (58.1%) as compared to 26 (41.9%) in those who received NACT. The majority of the patients (56.6%) had positive lymph nodes in both the pelvic and retroperitoneal groups, whereas 19.3% had only pelvic nodes positive, and 24.2% had only retroperitoneal nodes positive. The complications related to nodal dissection in our patients have been depicted in Table 3. A total of 15 patients had complications in our study. Six patients had intraoperative complications like pelvic splanchnic nerve injury and vascular injuries, which were tackled intraoperatively. Five patients developed chyle leaks, and two of these further developed lymphoceles which required re-admission and pigtail drainage. The other two patients with chyle leaks developed chylous ascites which were managed conservatively with intermittent drainage. Four other patients developed lymphoceles but they did not require any pigtail drainage and were managed with single-time ultrasound-guided aspiration.

**Table 1:** Clinicopathological characteristics of the patients

Characteristics	Total patients, *n* (%) (*n* = 105)
Age–years [median (range)]	51 (19–71)
ECOG performance status—no. (%)	
0–1	85 (80.9%)
≥ 2	20 (19.1%)
Duration of symptoms—months (range)	4.1 (1–13)
Histologic type—no. (%)	
Serous	86 (81.9%)
Mucinous	9 (8.6%)
Clear-cell	2 (1.9%)
Endometrioid	3 (2.8%)
Undifferentiated	2 (1.9%)
Mixed	3 (2.8%)
Histologic grade—no. (%)	
Well differentiated	17 (16.2%)
Moderately differentiated	58 (55.2%)
Poorly differentiated	30 (28.6%)
Stage (FIGO)	
III	69 (65.7%)
IV	36 (34.3%)
Upfront surgery	45 (42.9%)
Neoadjuvant chemotherapy	60 (57.1%)
Surgical PCI	
Less than 20	88 (83.8%)
More than 20	17 (16.2%)
Cytoreductive scores	
CC-0	83 (79%)
CC-1	22 (21%)
Nodal involvement	
Median LNs dissected (range)	15 (7–36)
Median Pelvic LNs dissected (range)	8 (6–18)
Median RP LNs dissected (range)	11 (9–19)
Bowel resection	15 (14.3%)
Upper abdominal surgical procedures	17 (16.3%)
Intraoperative blood transfusions	20 (19.0%)
Duration of surgery (min)	330 (240–540)
Mean ICU Stay (days)	1.62
Mean duration of hospital stay—days	8.54 ()
Recurrence	40 (38%)

*Abbreviations*: *PCI* Peritoneal Cancer Index, *LNs* lymph nodes, *RP* retroperitoneum

**Table 2:** Characteristics of the lymph nodes positive cases

Characteristics	Total cases, *n* (%)
Total LNs positive	62 (59%)
Upfront surgery	36 (58.1%)
Neoadjuvant chemotherapy	26 (41.9%)
Location of LNs involvement	
Only pelvic LNs	12 (19.3%)
Only RP LNs	15 (24.2%)
Both pelvic and RP LNs	35 (56.5%)
Number of LNs involvement	
01–10	42 (67.8%)
10–20	13 (20.9%)
> 20	7 (11.3%)

*Abbreviations*: *LN* lymph nodes, *RP* retroperitoneum

**Table 3:** Post-surgical complication rates related to lymphadenectomy in the patients

	HIPEC cases, *n* (%)	Non-HIPEC cases, *n* (%)
Total cases	45 (42.8%)	60 (57.2%)
Upfront surgery	12 (26.6%)	33 (55%)
Post-NACT	33 (73.4%)	27 (45%)
LNs positivity	26 (57.7%)	36 (60%)
Recurrence	11 (24.4%)	29 (48.3%)

In our study, 45 patients (42.8%) received HIPEC, among which 26 patients (57.7%) had lymph node positivity (Table 4). Sixty patients (57.2%) did not receive HIPEC, amongst which 36 patients (60%) had lymph node positivity. The recurrence rates were 24.4% (11) with HIPEC and 48.3% (29) in patients who did not receive HIPEC with a median follow-up of 36 months.

**Table 4:** Nodal positivity and recurrences in HIPEC and non-HIPEC cases

Characteristics	Total cases, *n* (%) *n* = 105
Intraoperative pelvic splanchnic nerve injury	3 (2.8%)
Intraoperative vascular injuries	3 (2.8%)
Chyle leak/lymphorrhea	5 (4.7%)
Lymphoceles	6 (5.7%)
Chylous ascites	4 (3.8%)
Readmission due to lymphocele	2 (1.9%)

*Abbreviations*: *NACT* neoadjuvant chemotherapy, *LNs* lymph nodes, *HIPEC* hyperthermic intraperitoneal chemotherapy

The lymph nodal positivity in our study as per each defined zone in the Nodal Cancer Index has been depicted in (Table 5). The most common sites of nodal positivity included Zone Rp 1, Rp2, and Rp3 which is the area above the inferior mesenteric artery (IMA).

**Table 5:** Nodal positivity in various NCI zones

Zones as per Nodal Cancer Index	Nodal positivity (%)
Zone Rp1	13%
Zone Rp2	19%
Zone Rp3	15%
Zone Rp4	9%
Zone Rp5	7%
Zone Rp6	9%
Zone P1	11%
Zone P2	7%
Zone P3	9%
Zone P4	13%
Zone P5	8%
Zone P6	10%
Zone 0^a^	2%

^a^Zone 0 includes positive lymph nodes in the inguinal region

**Fig. 2:**
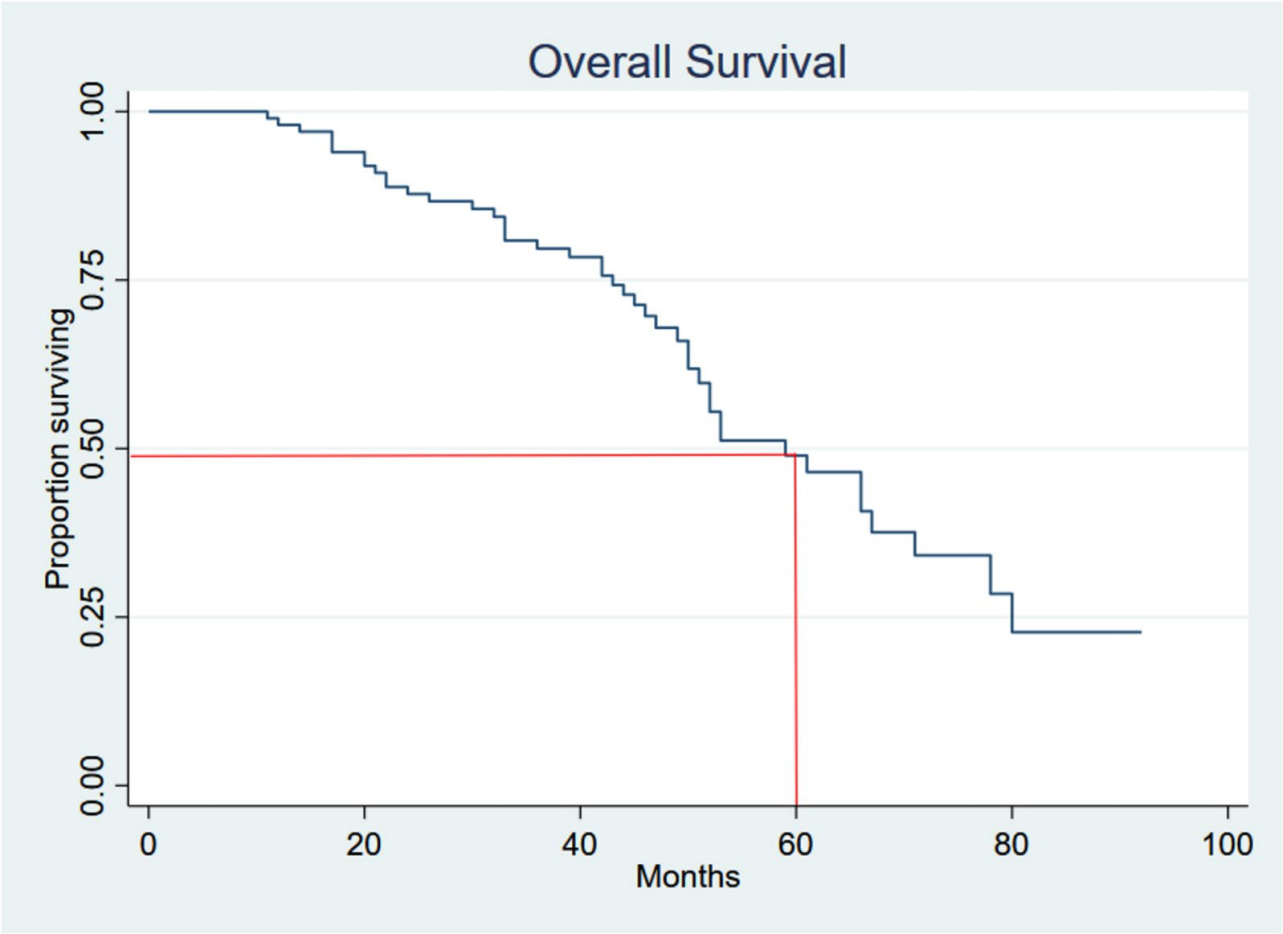
Overal survival in the patients

**Fig. 3:**
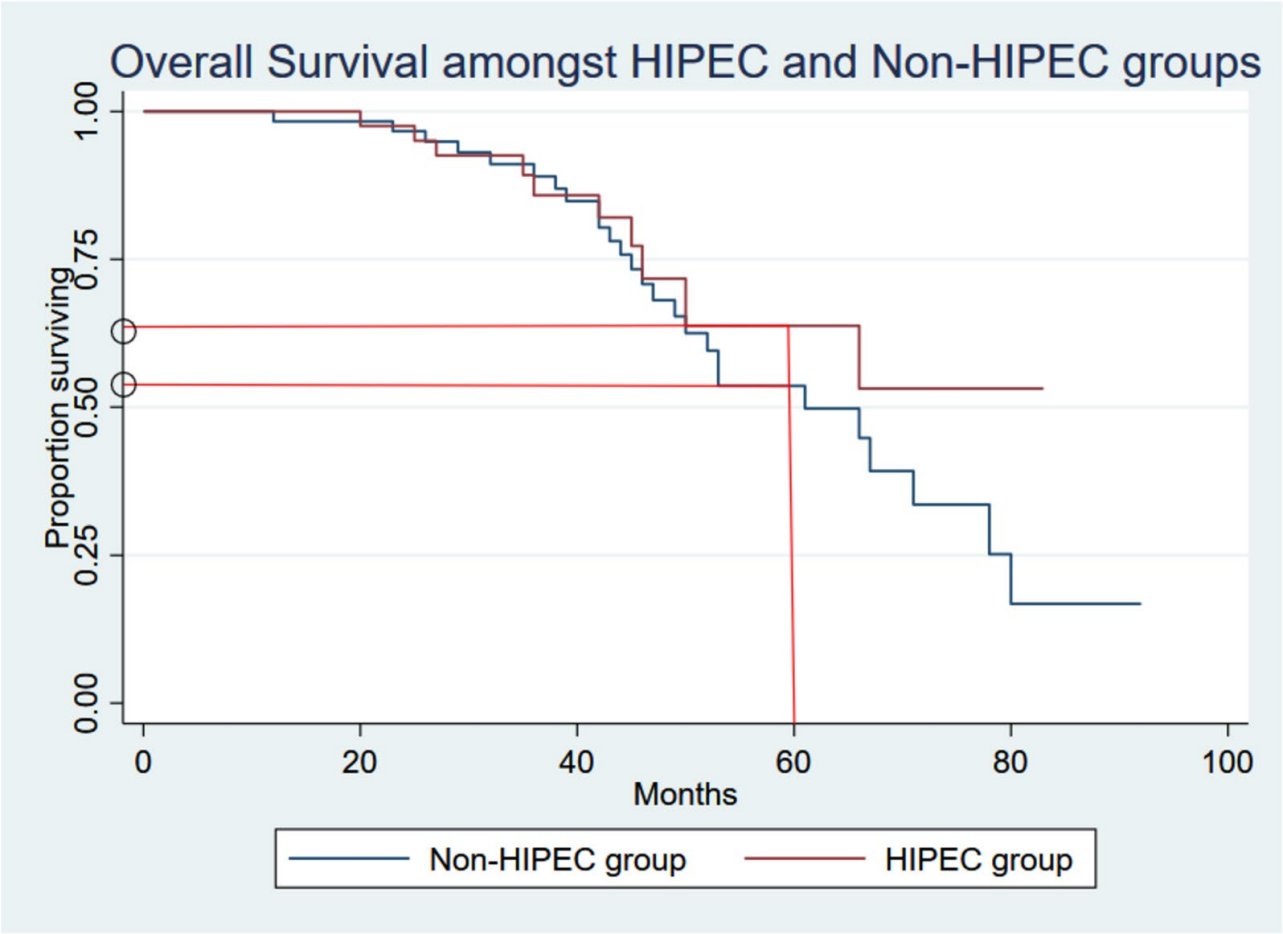
Overall survival in the HIPEC and non-HIPEC group

The probability of overall survival at 5 years in our patients was 48.9% (95% CI = 35.5–61) (Fig. 2). We compared the overall survival probability in patients who underwent HIPEC with those who did not undergo HIPEC. The median OS in the HIPEC group was 65 months [95% CI = 52.2–80.3], and in the non-HIPEC group was 61 months [95% CI = 45.6–76.3]. There was a non-significant difference in the probability of OS at 5 years in both groups. The probability of OS at 5 years in the HIPEC group was 63.7% [95% CI = 38.4–80.9] and in the non-HIPEC group was 53.5% [95% CI = 36.9–67.6] (Fig. 3).

## Discussion

The standard of care for advanced ovarian cancer remains CRS, followed by adjuvant chemotherapy. A complete optimal cytoreduction that removes all the macroscopic peritoneal disease burden remains the most important independent prognostic factor predicting survival in advanced ovarian cancer [6–8]. Peritoneal cancer index (PCI) has been described as a consistent predictor of the completeness of cytoreduction, but its role as an independent predictor of survival in ovarian cancer cases is still under consideration. Jacquet and Sugarbaker introduced [9, 10] The Peritoneal Cancer Index (PCI) in 1996 for peritoneal carcinomatosis of colorectal cancer and mesothelioma [11]. A consensus on a cutoff value for unresectability has not been established, but it does guide us to assess the burden of the disease, the extent, the prognosis, and the resectability rates uniformly [12]. However, the assessment of the PCI does not include the burden of the nodal status in the retroperitoneum and the pelvis.

In a single institutional study done in 1986, Burghardt et al. demonstrated a significant involvement of lymph nodes in stage III ovarian cancer, up to 61.8% in the total cohort and 75% even after chemotherapy, and also reported a survival benefit of systematic aortic and pelvic lymphadenectomy in advanced ovarian cancer. In this study, the aortic node positivity was 41.4%, and 5-year survival for stage III disease was 53% after lymphadenectomy compared to 13% without lymphadenectomy cases [13]. Overall previous studies showed the rate of involvement of lymph nodes was 45–60% in advanced ovarian cancer [14, 15]. In our analysis, total nodal positivity was 59% and 58.1% nodal positivity in upfront cases and 41.9% in post-NACT cases. Isolated retroperitoneal lymph nodes were positive in 24.2%, and isolated pelvic lymph nodes were positive in 19.3%.

In the guidelines, lymphadenectomy is recommended as a fundamental part of the surgical staging for all ovarian cancer patients that will guide the patient’s adjuvant therapy. In 1995, Spirtos et al. demonstrated the benefit of removing bulky lymph nodes, but there was no apparent benefit in resecting clinically negative lymph nodes [16]. Later, in 2005, a randomized control trial by Hacker et al. comparing systematic aortic and pelvic lymphadenectomy with resection of bulky nodes highlighted that overall survival is unaffected by systematic lymphadenectomy, but it did improve the progression-free survival [15]. Several retrospective studies have demonstrated an added survival advantage of systematic lymphadenectomy even in clinically negative lymph nodes [17–20]. In 2012, Chang et al., in a retrospective study of 189 patients, demonstrated the survival benefit of systematic lymphadenectomy in stage III ovarian cancer patients without any grossly visible disease [21]. However, the LION trial, a randomized control trial published in 2019, showed that removing nodes negative clinically is not indicated; instead, it increases morbidity and even mortality. Surprisingly, the rate of microscopic positive nodes was 55.7% in the lymphadenectomy arm which is a significant finding [22]. Apart from this, it is relevant to mention that the LION trial did not include patients who underwent interval debulking; thereby, it is not logical to conclude so far. Cornelis et al. showed that survival was significantly positively affected by various histologies with positive nodes. This study showed a significant effect on overall survival in serous ovarian cancers with nodal metastases compared with node-negative patients [4].

Various studies demonstrated that chemotherapy itself does not sterilize the involved nodes, and thereby these positive nodes become the potential sites for relapse, i.e., lymph nodes act as a “safe-haven” [23–26]. Nodal involvement post-chemotherapy, therefore, could be a harbinger or may be an indicator of the aggressiveness of the disease. Some studies have demonstrated the survival advantage of systematic lymphadenectomy in post-chemotherapy scenarios. In a Surveillance, Epidemiology, and End Results (SEER) data analysis by Chan et al., 13,918 patients with advanced-stage IIIC-IV were analyzed [17]. This study demonstrated the extent of lymphadenectomy to be a significant independent prognostic factor and was associated with improved disease-specific survival. In 2017, a study comparing lymph node sampling and lymph node dissection by Eoh et al. demonstrated lymph node dissection as an independent prognostic factor for overall survival as well as progression-free survival in post-neoadjuvant chemotherapy [27]. However, some studies did not show any added survival advantage of systematic lymphadenectomy in interval debulking cases [28, 29].

However, the optimal clearing of retroperitoneal nodes is technically challenging, especially after NACT. Even radiologically undetectable lymph nodes may be positive in around 40% of post-NACT cases as per our experience in advanced epithelial ovarian cancer. In node-negative cases, minimal sampling of the nodes should be done to complete the nodal staging, particularly from the para-aortic area above IMA, which is the most frequently involved site in 45% of the cases and considered the high alert zone as described in the previous work of the author [5]. The hypothesis of “safe haven” for metastatic ovarian cancer cells in retroperitoneal nodes even after NACT holds accurate thereby, systemic lymph node dissection should be continued until more prospective data is available against it [30]. Lymph nodal involvement in ovarian cancer is a poor prognostic factor, but genuinely speaking, there is no direct association of nodal positivity with disease recurrence or overall survival as cancer cachexia and intestinal obstruction prevail over the nodal burden.

### Proposed Nodal Cancer Index (NCI)

We strongly believe that accurate surgical staging, i.e., CRS, should include systemic lymphadenectomy in advanced ovarian cancer stage III onwards, which could detect the true extent of the disease as nodes are “safe haven” of micrometastasis. The overall effect of systemic lymphadenectomy on PFS is well-studied. Systematic lymphadenectomy is a favorable prognostic factor for advanced ovarian cancer.

We performed systematic lymph node dissection in all our advanced cases. We want to propose the NCI for a better understanding of patterns of lymph node involvement and better dissection like in left-sided tumor–para-aortic clearance, particularly above IMA, is first echelon lymph nodes as in 38% of cases, the initial involvement is in the nodes at this area which we had described as a high alert zone [5]. On the other hand, inter-aortocaval node involvement is the 1st echelon lymph node in 23% of the right-sided tumors [31]. In cases where sampling is indicated—at minimum, it should be done from these areas.

Now the question arises when HIPEC is to be performed. The essential requirement is optimal cytoreduction or minimal CC1 (nodule up to 2.5 mm). However, when the lymph nodes are not appropriately dissected or are unable to be dissected because of adherent to main vessels like the aorta, IVC, or renal vessels, should HIPEC be performed? When we declare—CRS has been achieved—should the lymph nodes also be cleared as part and parcel of the disease before HIPEC?

If in an optimally cytoreducted patient, the enlarged para-aortic lymph nodes or matted sheet-like lymph nodal mass is left behind, the whole idea of removing all visible diseases and gaining the advantage of HIPEC in such patients is lost. In the postoperative period and immediate follow-up, these visible and gross disease regions, even after undergoing extensive surgery and innovative HIPEC, haunt the patients, and their fear of disease never vanishes as ovarian cancer is one of the known notorious cancers and the recurrence rate is around 70% despite the multimodality treatment and mainly nodal and peritoneal recurrence. The whole scenario aggravates the already depressed patients. This impact of residual lymph nodes is very difficult to prove in our studies. The quality of life affected due to residual disease is an essential factor in managing these advanced ovarian cancer cases. Also, in the patients, the essential role of residual lymph nodes on survival cannot be accurately assessed in the studies. These patients succumb to subacute intestinal obstruction leading to intestinal failure and cancer cachexia. The residual lymph nodal burden in these cases cannot be well studied and its overall impact on survival as in the natural history, by the time the nodal disease is aggressive, the patients usually succumb to the disease.

Therefore, in our viewpoint, NCI is as crucial as PCI in such cases. It will help better document the lymph nodal burden and guide us in technically challenging successful lymph node dissection. In stage III–IV disease, cytoreduction should theoretically include all 13 zones to remove all evidence of tumor sites. As per the severity of NCI, the chance of nodal recurrence, and poor prognostic indicator, we divided the nodal burden as (i) high burden if NCI > 20, (ii) moderately burden if NCI = 10–20, low burden if NCI < 10.

Our study is retrospective in nature and includes ovarian cancers of serous histology. The numbers are low, and the disease burden in our cases could not be assessed uniformly for the analysis. Though there are a few limitations in our study, the NCI will help us to compare our data better in future retrospective as well as prospective trials. We are conducting a feasibility study to assess the impact of NCI on our cytoreductive score if lymph nodes are also included as part of it.

## Conclusion

- The results of our analytic observation confirm that systemic lymphadenectomy of all 13 zones proposed by this study should be an integral part of optimal CRS in the advanced carcinoma ovary. Some questions are still to be answered-
  1. The clinical assessment and the skill to remove all the enlarged nodes, especially pre-caval, para-caval, inter-aortocaval, and para-aortic areas, especially above the IMA.
  2. Without removing all these lymph nodes, should it be called optimal cytoreduction?
  3. Should HIPEC be used without removing the involved node that may be just a single node densely adherent to the great vessel wall?
  4. What should be other strategies without removing lymph nodes to address the lymph nodes, as our study showed 41.9% of patients had nodes positive after NACT?

- Although there is no direct, high-level evidence supporting overall survival, we experienced that any form of recurrence may decrease overall survival, even if it affects the patient’s mental status, which is hard to prove in a prospective study. However, with our proposed nodal cancer index and its applicability in all advanced ovarian cancer patients, we will be able to assess and manage these advanced cases in a better objective manner.

## Data Availability

The datasets used and/or analyzed during the current study are available from the corresponding author upon reasonable request.
